# Jinmaitong, a Traditional Chinese Compound Prescription, Ameliorates the Streptozocin-Induced Diabetic Peripheral Neuropathy Rats by Increasing Sciatic Nerve IGF-1 and IGF-1R Expression

**DOI:** 10.3389/fphar.2019.00255

**Published:** 2019-03-29

**Authors:** Wei Song, Wen Jiang, Chao Wang, Jun Xie, Xiaochun Liang, Ying Sun, Liyun Gong, Wei Liu, Ling Qu

**Affiliations:** ^1^Department of Traditional Chinese Medicine, Peking Union Medical College Hospital, Peking Union Medical College, Chinese Academy of Medical Science, Beijing, China; ^2^Center for Translational Medicine, Peking Union Medical College Hospital, Peking Union Medical College, Chinese Academy of Medical Science, Beijing, China

**Keywords:** diabetic peripheral neuropathy, Jinmaitong, IGF-1, IGF-1R, sciatic nerve

## Abstract

Jinmaitong (JMT) is a Traditional Chinese Compound Prescription for the treatment of diabetic peripheral neuropathy (DPN). This study aims to investigate the effect of JMT on the insulin-like growth factor 1 (IGF-1) and the insulin like growth factor 1 receptor (IGF-1R) expression in sciatic nerves of diabetic rats. Firstly, the chemical profile of JMT was characterized by UPLC/Q-TOF-MS analysis. A total of 72 compounds were putatively identified. Secondly, streptozotocin (STZ)-induced diabetic rats were treated with neurotropin (NTP, 2.67 NU/kg/day) or JMT at low-dosage (0.4375 g/kg/day), medium-dosage (0.875 g/kg/day), and high-dosage (1.75 g/kg/day) for continuous 16 weeks. Blood glucose and body weight were detected every 4 weeks during the experiment. The mechanical pain and morphological change on sciatic nerves were detected by pain measurement instrument and microscopy. The IGF-1 level in serum and tissues were measured though ELISA and immunohistochemistry. The mRNA and protein expressions of IGF-1, IGF-1R, peripheral myelin protein zero (P0), and peripheral myelin protein 22 (PMP22) in the tissues were measured by qRT-PCR and western blot. As a result, JMT had no significant effect on body weight, but reduced the fasting blood glucose levels of diabetic rats. Besides, the pathological morphology, mechanical pain thresholds, serum level and tissue expression of IGF-1, mRNA, and protein levels of IGF-1R, P0, and PMP22 were significantly improved in JMT group at middle dosage. In conclusion, JMT could ameliorate the behavioristics and morphology changes in DPN rats by promoting IGF-1 and IGF-1R gene and protein expressions in sciatic nerves, as well as regulating the peripheral nerve remyelination genes P0 and PMP22 expressions, which provides scientific evidence for the clinical application of JMT in DPN patients.

## Introduction

Diabetic peripheral neuropathy (DPN), the main type of diabetic neuropathy, is one of the most common chronic complications of diabetes. Studies show that over 50% of diabetics were eventually diagnosed with neuropathy, and about 1/4 of the diabetics suffered from chronic neuralgia, which severely affects the life quality of patients (Quattrini and Tesfaye, 2003). Compared with other diabetic complications, such as diabetic retinopathy and diabetic cardiomyopathy, DPN has the early-onset, and high-incidence characteristics. Typical pathological manifestations of DPN include aregeneratory and demyelination of myelinated nerve fibers, axonal dislocation, neuronal degeneration, and regeneration delay (Zenker et al., 2013). As a unique structure of the nervous system, myelin sheaths not only participate in the transmission of nerve electrical signals, but also have multiple effects such as nutrition, insulation, and protecting axons from the external environment damage. Because of the existence of myelin sheath, the axon between the node of Ranvier is isolated from the outside world, the foundation for the fast jumping conduction of nerve impulse. Therefore, the conduction speed of myelinated nerve fibers is faster than the unmyelinated. However, the myelinated nerve fibers are vulnerable. Once they are injured, it will take a long time for remyelination and repair, which is the main difficulty for nerve regeneration in DPN.

The insulin-like growth factor 1 (IGF-1), a kind of active protein polypeptide, is the product of autocrine and paracrine by a variety of cells in the body. Studies showed that IGF-1 could promote axonal growth, reduce neurons apoptosis, promote Schwann cells (SCs) proliferation, and migration, which is a kind of myelin formation in cells exclusively existing in the peripheral nerve system, and promote myelin regeneration (Piao and Liang, 2012). IGF-1 plays a neuroprotective role through binding to Insulin like growth factor 1 receptor (IGF-1R) and further activates the downstream myelination related genes. Peripheral myelin protein zero (P0) is a kind of glycoprotein which exclusively belongs to and constitutes the peripheral myelin sheath structure. P0 accounts for over 50% of the total protein in mature myelin organization, which is vital to form and maintain the normal myelin structure (Melcangi et al., 2000). Studies found that P0 gene was up-regulated in the process of myelin formation, suggesting that it played an important role in promoting myelin regeneration and repair (Tzakos et al., 2005). Peripheral myelin protein 22 (PMP22) is a membrane protein, highly expressed in the early stage of myelin formation, suggesting that PMP22 may mediate the adhesion between axons and SCs, which is one of the initial factors of myelin formation (Ohsawa et al., 2006). P0 and PMP22 are highly significant proteins in the constitution of myelin, which is crucial in the process of start, maintenance, and maturation of myelin (Lemke and Axel, 1985; Sakamoto et al., 1987; Notterpek et al., 1999; Melcangi et al., 2000; Tzakos et al., 2005; Ohsawa et al., 2006).

So far, there are few effective drugs for the treatment of DPN. As a non-protein extract from inflamed rabbit skin inoculated with vaccinia virus, Neurotropin® (NTP) has been widely used in Japan and China as an analgesic drug for treatment of chronic pain and peripheral inflammation, which has been used as a basic clinical treatment for DPN patients (Ning et al., 2004). Although NTP have an effect on ameliorating diabetic neuralgia and numbness, the treatment effect cannot be maintained for a long time and causes side effects such allergies and disorders in digestive system or the nervous system (Sakai et al., 2018). Therefore, it is of great significance to explore effective treatments for the prevention and treatment of DPN.

JMT is Traditional Chinese Compound Prescription used at the Peking Union Medical College Hospital (PUMCH), which showed obvious clinical effects on prevention and treatment of DPN for more than 10 years. It composed of 12 crude drug materials, including 10 herbs and two kinds of animal drugs. Our previous study showed that JMT could significantly improve the clinical symptoms such as numb, cold and pain in DPN patients, promote blood glucose and lipid metabolism, and accelerate nerve conduction velocity, evidenced by clinical double blind randomized controlled studies (Liang et al., 1999; Yin et al., 2015). Besides, it has been verified that JMT could alleviate the injury of peripheral nerve by improving the nerve conduction velocity, pain, and temperature sensation of DPN rats (Qu et al., 2008a; Shi et al., 2013; Yin et al., 2015). The possible mechanism may be related to reduce oxidative stress, increase the expression of nerve growth factor, ciliary neurotrophic factor, and BCl-2, decrease the expression of receptor of AGEs, nicotinamide adenine dinucleotide phosphate oxidase P22-phox subunit and caspase-3, reduce apoptosis, induce autophagy, as well as increase the proliferation activity of SCs (Qu et al., 2008a,b). These results are consistent with most of the modern pharmacological research, which has demonstrated that the herbs in the prescriptions such as Semen Cuscutae and Fructus Ligustri Lucidi could improve lipid peroxidation and inhibit the apoptosis of nerve cells. It has been shown that autophagy was induced by quercetin, which is the effective ingredients of Semen Cuscutae, and other antioxidants (Liu et al., 2011; Wu et al., 2011);(Yan et al., 2012).

Despite this, the chemical composition and the pharmacological mechanism of JMT are poorly understood. Particularly, the relationship between JMT and the IGF-1 and IGF-1R still remains unknown. The present study aims to identify the main constituents in JMT and investigate the effect of JMT on specific markers of myelin sheath P0 and PMP22, as well as insulin growth factors involved in demyelination and remyelination, such as IGF-1 and IGF-1R in the sciatic nerve of a diabetic rat model.

## Materials and Methods

### Drug Materials and Sample Preparation for UPLC/MS Analysis

JMT is composed of 12 crude drug materials, including the seeds of *Cuscuta chinensis* Lam., the seeds of *Ligustrum lucidum* Ait., the herb of *Eclipta prostrata* L., the herb of *Prunella vulgaris* L., the seeds of *Litchi chinensis* Sonn., *Buthus martensii* K., the tender stem of *Cinnamomum cassia* Presl., the rhizoma of *Corydalis yanhusuo* W. T. Wang, the seeds of *Prunus persica* L., the seeds of *Cassia obtusifolia* L, the radix and rhizoma of *Asarum heterotropiodes* F., and *Hirudo nipponica* W. as a fixed ratio of 10:10:10:10:30:3:10:10:10:30:3:3. These crude drugs were bought from Tong Ren Tang Lit. Corp (Beijing, China) and authenticated by Prof. X.C. Liang. Detailed information of the drug materials and the scan of the vouchers were given in Supplementary Table 1. The voucher specimens (No. jmt15A–jmt15L) were deposited at the Department of Traditional Chinese Medicine, Peking Union Medical College Hospital, Beijing, China. After grounding into powder and homogeneous mixing, an aliquot of 100 mg of the JMT powder was extracted in 1 mL of water and 1 mL of methanol, respectively. The two supernatants were combined and filtered through a 0.2 μm membrane before use.

### UPLC/Q-TOF-MS Analysis

UPLC/MS analysis was performed on a UPLC system coupled with XEVO G2 Q-TOF mass spectrometer via an ESI source (Waters Corp., Milford, MA). For UPLC separation, 2 μL of sample solution was injected into an ACQUITY HSS T3 C_18_ column (100 × 2.1 mm, 1.7 μm, Waters). The mobile phase consisted of ACN (A) and water containing 0.1% (v/v) formic acid (B). Linear gradient elution was applied (0–5 min, 5–30% A; 5–10 min, 30–40% A; 10–20 min, 40–65% A; 20–25 min, 65–90% A) at a flow rate of 0.4 mL/min. The column temperature was 45°C. For MS detection, accurate mass was maintained by the LockSpray interface of sulfadimethoxine (309.0658 [M-H]^−^). The operating parameters in negative ion mode were as follows: capillary voltage, 3.0 kV; cone voltage, 30 V; desolvation gas flow rate, 750 L/h; source temperature, 120°C; desolvation temperature, 350°C. MS data were acquired in centroid mode and processed by MassLynx software (Waters, version 4.1).

### Animals

Male 6-week-old Sprague Dawley rats (*n* = 60; body weight, 160–200 g) were provided by Vital River Laboratory Animal Technology Co., Ltd. [Beijing, China; certificate no. SCXK (Beijing) 2011-0004], and were bred in the Experimental Animal Center (specific pathogen-free level) of the Peking Union Medical College Hospital (Beijing, China). Rats were acclimated for 1 week before the commencement of the experiment. This study was carried out in strict accordance with the recommendations of the Guide for the Care and Use of Laboratory Animals of the National Institutes of Health. The protocol was approved by the Institutional Animal Care and Use Committee of the Peking Union Medical College Hospital.

### Diabetic Rat Model and Grouping

The diabetic rat model was induced as previously described (Kim et al., 2000). Briefly, after a 1-week adaptation period, rats were randomly divided into the normal and diabetic model (DM) groups. After a 12 h fast, a 0.45% streptozotocin (60 mg/kg) solution (Sigma, St. Louis, MO, USA) prepared in 0.1 mol/L citrate buffer (pH 4.5), was intraperitoneally injected in rats of the DM group, while rats in the normal group were injected with the same volume of 0.1 mol/L citrate buffer. Blood glucose was measured from the tail tip 72 h later using a blood glucose meter (MediSense® Optium™; Abbott Laboratories, Chicago, IL, USA). Blood glucose levels ≥16.7 mmol/L indicated model success. Consequently, 50 successfully modeled diabetic rats were randomly divided into five groups (*n* = 10/group): DM control, JMT low-dosage (JMT-L), JMT medium-dosage (JMT-M), JMT high-dosage (JMT-H), and Neurotropin (NTP). The normal control group (Con) included 10 rats with blood glucose levels <7.0 mmol/L.

### Drug Administration

Following model success, the dosage for JMT-L, JMT-M, and JMT-H groups were 0.4375, 0.875, and 1.75 g/kg/day, respectively. The doses were calculated based on the well-mixed JMT powders. The NTP group received 2.67 NU/kg/day neurotropin (4.0 NU/tablet, Nippon Zoki Pharmaceutical Co Ltd, Osaka, Japan; lot no.S20090094). These drugs were prepared in distilled water. The DM and Con groups were orally fed distilled water (10 mL/kg/day). The rats were treated for 16 weeks. Body weight and tail tip fasting blood glucose levels were determined prior to drug administration, and at weeks 4, 8, 12, and 16.

### Mechanical Pain Threshold

After 16 weeks of treatment, prior to the sacrifice of the rats, mechanical pain threshold assessment was performed using the Von Frey Pain Measurement Instrument (cat. no. 2391; IITC Life Science Inc., Woodland Hills, CA, USA). The rats were placed in an elevated metal net, and covered with transparent organic glass. After a 15-min adaptation period, the middle of the hind foot of each rat was vertically stimulated with the electronic Von Frey probe, making it appear slightly S-shaped, and the paw withdrawal response was observed. A quick flinching reaction immediately subsequent to stimulation was considered to be a positive reaction, and the values (g) were recorded. A paw withdrawal reaction caused by physical activity was not reported as positive.

### Blood Collection and Tissue Specimen

All rats were sacrificed after 16 weeks of treatment. The rats were intraperitoneally injected with 10% chloral hydrate (3 mL/kg). Blood samples were collected from the carotid artery. After standing at room temperature for 2 h, they were centrifuged at 4,000 r/min for 10 min. Serum were stored at −80°C. After blood collection, the bilateral sciatic nerves were cut with sharp scissors and rinsed with ice-cold phosphate saline buffer immediately. The left sciatic nerve was isolated and cut into two segments: one segment (2 mm) was fixed in 2.5% glutaraldehyde for ultrastructure observation; the other segment (1 cm) was fixed in 4% PFA for pathological and immunohistochemical staining. The remaining segment and the right sciatic nerve were immediately frozen in liquid nitrogen and stored at −80°C.

### Electron Microscopy

Sciatic nerves were fixed in 2.5% glutaraldehyde 2–4 h. Nerves were rinsed in PBS, post fixed in 1% osmium tetroxide for 2 h, dehydrated in a graded ethanol series, infiltrated with propylene oxide, and embedded in epon. Ultrathin sections (60–80 nm) were stained with uranyl acetate and lead citrate. The myelin sheaths were observed by using a transmission electron microscope (TEM-1400 plus, JEOL, Tokyo, Japan) at 10,000 × and 15,000× magnification.

### Sciatic Nerve Histology and Immunohistochemistry

The 4% paraformaldehyde fixed sciatic nerve tissues were embedded in paraffin, and then cut at a thickness of 5 μm. Sections were stained with hematoxylin and eosin, and were imaged with a light microscope (CI-S, Nikon, Tokyo, Japan). The other sciatic nerve sections were treated with the following steps: regular dewaxing, repair of hot antigens with citric acid buffer (pH 6.0), blockade of endogenous peroxidase with 3% H_2_O_2_. The sections were incubated overnight with primary antibody IGF-1 (1:100; Bioword, Minnesota, USA) at 4°C, with HRP Detection Systems, respectively, and then developed with diaminobenzidine (DAB) kit. (detailed information of antibodies was shown in Supplementary Table 2). The sections were then observed using a Leica DM3000 microscope and the Leica image acquisition system (Leica Microsystems, Wetzlar, Germany). Image-Pro Plus 6.0 software (Media Cybernetics, Inc., Rockville, MD, USA) was used for image analysis. Immunohistochemistry results were measured using the integrated optical density.

### Enzyme-Linked Immunosorbent Assay (ELISA)

Serum IGF-1 was measured with commercially available ELISA kit (eBioscience, California, USA) according to the manufacturer's instructions. Standard or sample, detection reagent, substrate solution, and stop solution will be sequentially added to wells and incubated with repeated washing as appropriate. Optical density at 450 nm was immediately measured with a plate reader, and sample values were then calculated from the standard curve.

### Real-Time Reverse Transcription-Polymerase Chain Reaction (RT-PCR) Assays

Total mRNA was extracted from sciatic nerve tissue using TRIzol (ThermoScientific, MA, USA), according to the manufacturer's instructions. RNA purity was determined using absorbance at 260 and 280 nm (A260/A280). Reverse transcription of RNA was conducted according to the manufacturer's instructions (TaKaRa, Dalian, China). The primer sequences are shown in Table 1. mRNA quantification was performed via qPCR using the ChamQ™ Universal SYBR® qPCR Master Mix (Vazyme Biotech Co., Ltd., Nanjing, China) in a LineGene real-time PCR detection system (Applied Biosystems, Thermo Fisher Scientific Co., Ltd., Waltham, MA, USA). The qPCR reaction program was as follows: Pre-incubation at 95°C for 5 min; amplification at 95°C for 10 s, 60°C for 60 s for 40 cycles; separation at 95°C for 10 s, 60°C for 60 s. After amplification, standard curves were generated for target genes, which were then normalized against an endogenous reference gene GAPDH. Relative expression of target genes was determined by the 2^−ΔΔ*Ct*^ method.

**Table 1:** The primer sequences for RT-PCR assay.

**Gene**		**Primer sequence (5^**′**^-3^**′**^)**
IGF-1	Forward	5′-AGCTGTGATCTGAGGAGGCT−3′
	Reverse	5′-CCTTTCCTTCTCCTTTGCAG−3′
IGF-1R	Forward	5′-CGAGACATCTACGAGACGGA−3′
	Reverse	5′-AAAGGACCAGACATCGGAAT−3′
P0	Forward	5′-GGACATAGTGGGCAAGAC−3
	Reverse	5′-AGGTAGAAGAGCAACAGCA−3
PMP22	Forward	5′-GTCCTGTCCCTGTTCCTGTT−3
	Reverse	5′-GCTGCACTCATCACACACAG−3
GAPDH	Forward	5′-CAAGTTCAACGGCACAGTCA−3
	Reverse	5′-ATCTCGCTCCTGGAAGATGG−3

### Western Blot Analysis

All the selected proteins extracts of sciatic nerves were resolved by 10% SDS-PAGE and transferred on PVDF (Millipore, Bedford, MA, USA) membranes. After blocking, the PVDF membranes were washed four times for 15 min with TBST at room temperature and incubated with primary antibodies overnight at 4°C. The following primary antibodies were used: Anti-IGF1 monoclonal antibody, Anti-P0 monoclonal antibody, Anti-PMP22 monoclonal antibody (all 1:1,000; Abcam, Cambridge, MA, USA), Anti-p-IGF1R polyclonal antibody (1:100; Abcam, Cambridge, MA, USA), and β-actin (1:10,000, Abcam, Cambridge, MA, USA). Following extensive washing, membranes were incubated with secondary horseradish peroxidase (HRP)-conjugated secondary antibodies (1:10,000, Abcam, Cambridge, MA, USA) for 4 h at room temperature (detailed information of antibodies was shown in Supplementary Table 2). After further washed with TBST, the immunoreactivity was visualized by enhanced chemiluminescence (ECL kit, Millipore, Billerica, MA, USA), and the membranes were exposed to KodakXAR-5 films. Relative optical density (ROD, ratio to β-actin) of each blot band was quantified by using National Institutes of Health (NIH) image software (Image J 1.36b).

### Statistical Analyses

SPSS 17.0 software (SPSS Inc., Chicago, IL, USA) was used for the statistical analysis. The one-sample Kolmogorov-Smirnov *Z*-test was used to determine if the variables were normally distributed. Normally-distributed data are expressed as the mean ± standard deviation. Multi-group independent samples were compared using one-way analysis of variance, with the least significant difference *post hoc* test. Non-normally-distributed data were analyzed with non-parametric tests. *P* < 0.05 was considered to indicate a statistically significant difference.

## Results

### Chemical Profiling of JMT

**Figure 1:**
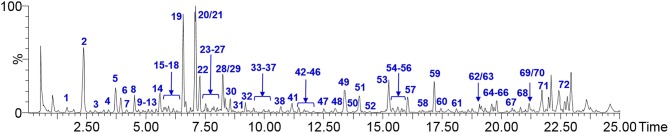
Base peak ion (BPI) chromatogram of JMT in negative scan.

UPLC/Q-TOF-MS analysis was employed to characterize the chemical composition of JMT. A total of 72 peaks (**1**–**72**) were putatively identified by comparing their high-resolution MS data with those in literature (He et al., 2011; Zhang et al., 2012; Li et al., 2014; Liu et al., 2014; Fang et al., 2015; Lv et al., 2015; An et al., 2017; Sandjo et al., 2017; Zhong et al., 2017; Zheng et al., 2018). These compounds have covered most of the main peaks in the chromatogram and different kinds of constituents were involved, such as flavone glycosides (e.g., 17, 20–22, 28–30), flavonoid aglycone (e.g., 26, 41, 49, 54, 59, 60), phenolic acids (e.g., 1–4, 15, 36), triterpenoids (e.g., 40, 64) and so on (Figure 1, Table 2). Among them, hyperin (21) and isoquercitrin (22) could be metabolized into quercetin (41) *in vivo*, which showed obvious anti-diabetes and anti-inflammation bioactivities (D'Andrea, 2015). Besides, there are also some polypeptides, amino acids, and polysaccharides, which mainly derived from the two animal drugs in JMT and not displayed in the present fingerprint.

**Table 2:** Characterization of chemical constituents in JMT by UPLC-QTOF-MS analysis.

**Peak No**.	**t_**R**_ (min)**	**Measured [M–H]^**−**^ (m/z)**	**Predicted [M–H]^**−**^ (m/z)**	**Δ (ppm)**	**Formula**	**(–)MS^**E**^ (m/z)**	**Identification**	**Derived from**
1	1.72	169.0130	169.0137	−4.14	C_7_H_5_O_5_	125.0235, 83.0116	Gallic acid	D
2	2.36	515.1127	515.1190	−12.23	C_25_H_24_O_12_	353.1021, 191.0067	Isochlorogenic acid C	C
3	2.79	167.0334	167.0342	−4.79	C_8_H_8_O_4_	135.0817	Vanillic acid	D
4	3.98	153.0180	153.0188	−5.23	C_7_H_6_O_4_	109.029	Protocaechuic acid	E
5	3.73	353.0997	353.1025	−7.93	C_20_H_18_O_6_	339.0869, 310.0527	Asarinin	J
6	3.85	633.3972	633.4003	−4.89	C_36_H_58_O_9_	471.3481, 453.3340	Ecliptasaponin A or D	C
7	4.23	633.3977	633.4003	−4.10	C_36_H_58_O_9_	471.3482, 453.3339	Ecliptasaponin A or D	C
8	4.50	289.0735	289.0712	7.96	C_15_H_14_O_6_	245.0803, 205.0510, 179.0345	Epicatichin	E
9	4.53	919.2710	919.2719	−0.98	C_39_H_52_O_25_	271.0615, 256.0369	Cassiaside B2	I
10	4.98	685.2333	685.2344	−1.61	C_31_H_42_O_17_	523.1820, 453.3307	Specnuezhenide	B
11	5.10	313.0352	313.0348	1.28	C_16_H_10_O_7_	313.0350, 298.1503	Wedelolactone	C
12	5.29	609.1882	609.1819	10.34	C_28_H_34_O_15_	447.1285, 285.0753	Hesperidin	E
13	5.47	901.2633	901.2614	2.11	C_39_H_50_O_24_	253.0511	Emodin-1-O-β-D-tetrapyranoglucoside	I
14	5.58	283.0221	283.0243	−7.77	C_15_H_8_O_6_	255.0287, 239.0338, 211.0173	Rhein	I
15	5.77	353.0856	353.0873	−4.81	C_16_H_18_O_9_	191.0565, 112.9657	Chlorogenic acid	A,C
16	5.80	957.5075	957.5059	1.67	C_48_H_78_O_19_	795.4522, 455.3437	Ecliptasaponin B or III	C
17	6.00	563.1412	563.1401	1.95	C_26_H_28_O_14_	473.1082, 443.0975, 383.0764	Schaftoside	E
18	6.16	340.1535	340.1549	−4.12	C_20_H_23_NO_4_	178.0552, 163.0738	Tetrahydrojatrorrhizine	G
19	6.62	595.1617	595.1663	−7.73	C_27_H_32_O_15_	271.0625, 255.0403	Rubrofusarin-6-o-β-gentiobioside	I
20	7.09	463.0883	463.0877	1.30	C_21_H_20_O_12_	463.0882, 301.0350	Hyperoside	A
21	7.15	419.1029	419.0978	12.17	C_20_H_20_O_10_	257.0465	Cassiaside	I
22	7.28	463.0888	463.0877	2.38	C_21_H_20_O_12_	463.0885, 301.0347	Isoquercitrin	A,C,D
23	7.51	253.0515	253.0501	5.53	C_15_H_10_O_4_	225.0110, 149.0025	Chrysophanol	C
24	7.65	491.1178	491.1190	−2.44	C_23_H_24_O_12_	329.0618, 298.0155	Hesperidin-6-o-β-D-glucoside	I
25	7.73	447.0923	447.0927	−0.89	C_21_H_20_O_11_	285.0411, 175.0387, 133.0280	Luteoline 7Oglucoside	A,D
26	7.83	465.1020	465.1033	−2.80	C_21_H_22_O_12_	303.0502, 285.0471	Dihydroquercetin	B
27	8.05	795.4479	795.4531	−6.54	C_42_H_68_O_14_	841.4561, 633.3463, 453.3351	Ecliptasaponin I,C,IV or XV	C
28	8.23	447.0932	447.0927	1.12	C_21_H_20_O_11_	285.0395, 175.0386, 151.0023	Luteoloside	I
29	8.23	445.0787	445.0771	3.59	C_21_H_18_O_11_	269.0461, 151.0023, 117.0315	Apigenin-7-O-glucronide	J
30	8.52	447.0934	447.0927	1.57	C_21_H_20_O_11_	285.0404, 175.0388, 133.0281	Astragalin	A
31	9.06	565.1522	565.1558	−6.37	C_39_H_52_O_25_	271.0609, 256.0385	Cassiaside B	I
32	9.25	685.2346	685.2344	0.29	C_31_H_42_O_17_	523.1809, 453.3298	Nuezhenoside	B
33	9.50	785.2531	785.2504	3.44	C_35_H_46_O_20_	623.1957, 477.1320, 299.0743	Echinacoside	B
34	9.64	843.4295	843.4378	−9.84	C_42_H_68_O_17_	841.4507, 633.3500, 471.3392	Ecliptasaponin VI	C
35	9.98	623.1966	623.1976	−1.60	C_29_H_36_O_15_	461.1445, 153.0761	Acteoside	B
36	10.12	359.0758	359.0767	−2.51	C_18_H_16_O_8_	322.1003	Rosmarinicacid	D
37	10.25	269.0463	269.0450	4.83	C_15_H_10_O_5_	241.0471, 213.0545, 185.086	Emodin	G
38	10.60	299.1139	299.1131	2.67	C_14_H_20_O_7_	137.0587, 119.0495	Salidroside	B
39	10.96	1071.3639	1071.3557	7.65	C_48_H_64_O_27_	1117.2851, 909.2393, 685.5023	Oleonuezhenide	B
40	11.00	453.3335	453.3369	−7.50	C_30_H_46_O_3_	437.3401, 411.3272	3-hydroxy oleanolic acid	C
41	11.16	301.0339	301.0348	−2.99	C_15_H_10_O_7_	181.0131, 165.9892, 119.0493	Quercetin	A,C,D
42	11.50	505.1331	505.1346	−2.97	C_24_H_26_O_12_	343.0829, 313.0352	Cassiin	I
43	11.63	449.1073	449.1084	−2.45	C_21_H_22_O_11_	287.0547, 151.0025,	Eriodictyol-7-o-glucoside	J
44	11.67	451.1072	451.1088	−3.55	C_17_H_24_O_14_	407.2264, 375.0891	Privet acid	B
45	11.86	1071.3564	1071.3557	0.65	C_48_H_64_O_27_	909.3015, 685.1958, 299.0939	Nuezhenide G13	B
46	12.00	368.1875	368.1862	3.53	C_22_H_27_NO_4_	336.1043, 320.1274, 294.0861	Corydaline	G
47	12.52	279.2330	279.2324	2.15	C_18_H_32_O_2_	279.2329	Linoleic acid	H
48	13.04	283.0623	283.0607	5.65	C_16_H_12_O_5_	268.0368, 240.0397	Obtusifolin	I
49	13.38	285.0370	285.0399	−10.17	C_15_H_10_O_6_	175.0392, 151.0018, 133.0279	Kaempferol	A
50	13.80	431.0983	431.1009	−6.03	C_21_H_19_O_10_	269.0451	Emodin-6-o-β-D-glucoside	I
51	14.00	343.0835	343.0818	4.96	C_18_H_16_O_7_	313.0328, 285.0362, 270.0164	Obtusin	I
52	14.55	357.1014	357.0975	10.92	C_19_H_18_O_7_	313.0279, 269.0085, 241.0153	Methyl obtusin	I
53	15.25	329.0652	329.0661	−2.74	C_17_H_14_O_7_	298.0123, 270.0167	Aurantio-obtusin	I
54	15.46	461.0711	461.0720	−1.95	C_21_H_18_O_12_	285.0410, 267.0309, 175.0237,	Scutellarin	J
55	15.59	519.1512	519.1503	1.73	C_25_H_28_O_12_	227.1255, 209.0870	6′-O-cinnamoyl-8-epikingisidic acid	B
56	15.83	149.0595	149.0597	−1.34	C_9_H_8_O_2_	149.0597, 131.0492	Cinnamic acid	F
57	16.12	461.0741	461.0720	4.55	C_21_H_18_O_12_	279.0408, 285.0025	Kaempferol-3-O-glucuronide	A
58	16.85	431.0962	431.0978	−3.71	C_21_H_20_O_10_	325.1591, 285.0389	Kaempferol 7-O-α-L-rhamnopyranoside	A
59	17.27	285.0382	285.0399	−5.96	C_15_H_10_O_6_	175.0391, 151.0022, 133.0279	Luteolin	A,C
60	17.50	269.0459	269.0450	3.35	C_15_H_10_O_5_	151.0020, 149.0228, 117.0321	Apigenin	A
61	18.12	539.1752	539.1765	−2.41	C_25_H_32_O_13_	377.1230, 275.0855	Oleuropein	B
62	19.18	287.0551	287.0556	−1.74	C_15_H_12_O_6_	151.0025, 135.0438	Eriodictyol	J
63	19.25	255.2331	255.2324	2.74	C_16_H_32_O_2_	255.2331	Hexadecanoic acid	H
64	19.57	455.3552	455.3525	5.93	C_30_H_48_O_3_	455.3551, 201.0361	Oleanolic acid or ursolic acid	B, D
65	19.70	131.0492	131.0497	−3.82	C_9_H_8_O	131.0492	Cinnamaldehyde	F
66	19.82	161.0594	161.0603	−5.59	C_10_H_10_O_2_	131.0493	2methoxycinnamaldehyde	F
67	20.50	413.3765	413.3783	−4.35	C_29_H_50_O	413.3768	β-sitosterol	H
68	20.91	471.3503	471.3474	6.15	C_30_H_48_O_4_	453.3360, 425.3397	Echinocystic acid	C
69	21.20	271.0620	271.0606	5.16	C_15_H_12_O_5_	151.0021, 119.0489	Naringenin	E
70	21.25	502.1558	502.1566	−1.59	C_20_H_27_NO_11_	295.1047, 133.0652	Amygdalin	H
71	21.73	411.3650	411.3627	5.59	C_29_H_48_O	411.3651, 397.3452	Stigmasterol	E
72	22.71	181.0495	181.0501	−3.31	C_9_H_9_O_4_	151.6605, 136.9081	Syringaldehyde	F

*A, seeds of Cuscuta chinensis Lam.; B, seeds of Ligustrum lucidum Ait.; C, whole herb of Eclipta prostrata L.; D, whole herb of Prunella vulgaris L.; E, seeds of Litchi chinensis Sonn.; F, tender stem of Cinnamomum cassia Presl.; G, rhizoma of Corydalis yanhusuo W.; H, seeds of Prunus persica L; I, seeds of Cassia obtusifolia L. or Cassia tora L.; J, radix and rhizoma of Asarum heterotropiodes F*.

### JMT Reduced the Fasting Blood Glucose Levels but Had No Effect on the Body Weight of DPN Rats at 16 Weeks

**Figure 2:**
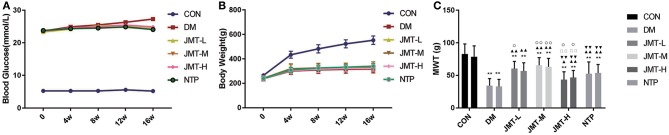
Effect of JMT on fasting blood glucose levels, body weight, and mechanical pain threshold in DPN rats. **(A)** Effect of JMT on fasting blood glucose levels in different groups in different time points. **(B)** Effect of JMT on body weight in different groups in different time points. **(C)** Effect of JMT on the left- and right- paw mechanical pain threshold in different groups at 16 w. **P < 0.01 vs. Con group; ^▴^*P* < 0.05 vs. DM group, ^▴▴^*P* < 0.01 vs. DM group; ^▾▾^*P* < 0.01 vs. JMT-M group; ^□□^*P* < 0.01 vs. JMT-L group; °*P* < 0.05 vs. NTP group, °°*P* < 0.01 vs. NTP group.

Fasting blood glucose levels in all the diabetic rats were higher than those in the control group rats at all time-points (Supplementary Table 3, *P* < 0.01). No significant differences in fasting blood glucose levels were found among the DM, NTP, and JMT groups at 4, 8, 12 weeks (Supplementary Table 3, *P* > 0.05). The fasting blood glucose levels in NTP and JMT groups were lower than DM groups at 16 weeks (Supplementary Table 3, *P* < 0.01) (Figure 2). No significant differences in body weight were observed at baseline among the different groups (*P* > 0.05). After 4 weeks of treatment, the body weight of the diabetic rats was lower than that of the control group rats (*P* < 0.01). No significant differences in body weight were found among the DM, NTP, and JMT groups at any time-points (Supplementary Table 4, *P* > 0.05) (Figure 2).

### JMT Increased the Mechanical Pain Threshold in DPN Rats

The mechanical pain threshold values are listed in Supplementary Table 5. Compared with the normal controls, the left mechanical pain threshold values were significantly lower in the DM group (*P* < 0.01). The left mechanical pain threshold values of each treatment group were higher than those of the DM group (*P* < 0.05). Among the treatment groups, the left mechanical pain threshold values were significantly higher in the JMT-M and JMT-L groups compared with that in the NTP groups (*P* < 0.05). JMT-H group was obviously lower than NTP group (*P* < 0.05). No significant differences in the left mechanical pain threshold values between JMT-M and JMT-L groups (*P* > 0.05) (Figure 2). Compared with the normal controls, the right mechanical pain threshold values were significantly lower in the DM group (*P* < 0.01). The right mechanical pain threshold values of each treatment group were higher than those of the DM group (*P* < 0.01). Among the treatment groups, the right mechanical pain threshold values were significantly higher in the JMT-M and JMT-L groups compared with that in the JMT-H groups (*P* < 0.01). JMT-M group was higher than NTP group (*P* < 0.01), while JMT-H group was lower than NTP group (*P* < 0.05). No significant differences in the right mechanical pain threshold values between NTP and JMT-L groups (*P* > 0.05) (Figure 2).

### JMT Improved the Neurological Morphology of Sciatic Nerve in DPN Rats

**Figure 3:**
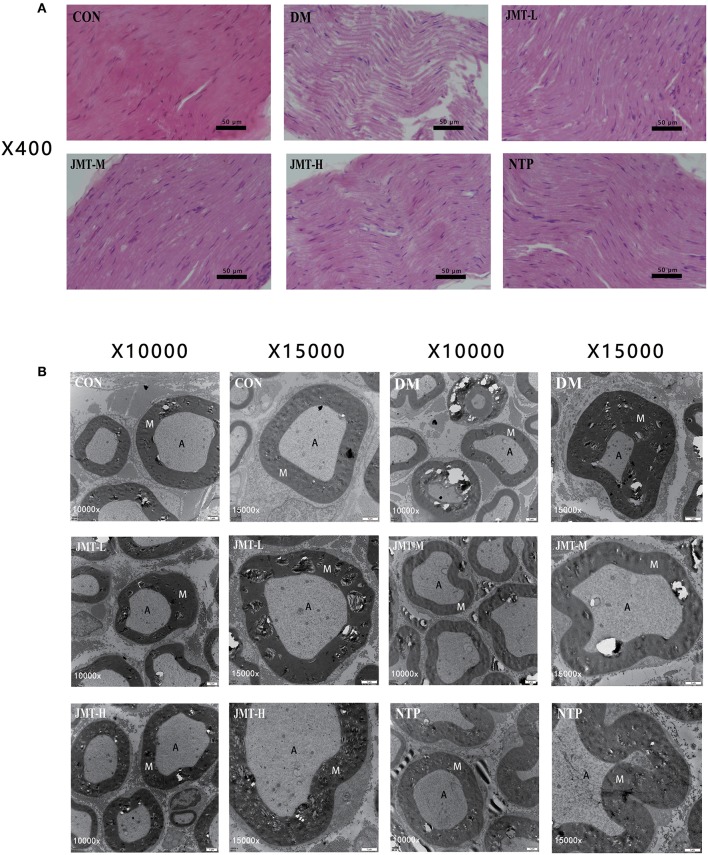
JMT improved the neurological morphology of sciatic nerve in DPN rats. **(A)** H&E staining of longitudinal section slices of sciatic nerve in Control, DM, JMT-L, JMT-M, JMT-H, and NTP groups, respectively. H&E staining images were viewed at a magnification of 400. **(B)** TEM of cross section slices of sciatic nerve in Control, DM, JMT-L, JMT-M, JMT-H, and NTP groups, respectively. TEM staining images were viewed at a magnification of 10,000 and 15,000.

Demyelination and axon atrophy of sciatic nerve were evaluated by H&E staining and ultrastructure observation using TEM. As shown in Figure 3A, H&E staining demonstrated that no histological damages occurred in the control group. The myelinated nerve fibers and sheaths were uniformly distributed. By contrast, in the model group, the myelinated nerve fibers were sparsely distributed, the thickness of sheaths was uneven, the vacuolization of nerve fiber with decreased myelinated was observed, while the degree of degeneration was ameliorated in the treatment groups. Meanwhile, representative ultrastructure of sciatic nerve demonstrated the regular myelin thickness and normal structure in control group while the myelin sheath cells became disordered in the modulated fibers of sciatic nerves in the DM groups. Shrinkage, deformation, and inhomogeneity were observed in neurites. The structures of SCs blurred and the vacuoles degenerated, but the integrity of cell membranes was not lost. Cells of non-myelinated nerves swelled, with irregular morphology, and widened cellular spaces. SCs shrank. In the treatment groups, the pathological structures of neurites and myelin were improved at different levels. Deranged myelin sheath cells recovered and SCs swelling was attenuated. The JMT treatment prevented the sheaths degeneration and improved the nerve fiber recovery (Figure 3B).

### JMT Raised the IGF-1 Expression in the Serum and Sciatic Nerve of the DPN Rats

**Figure 4:**
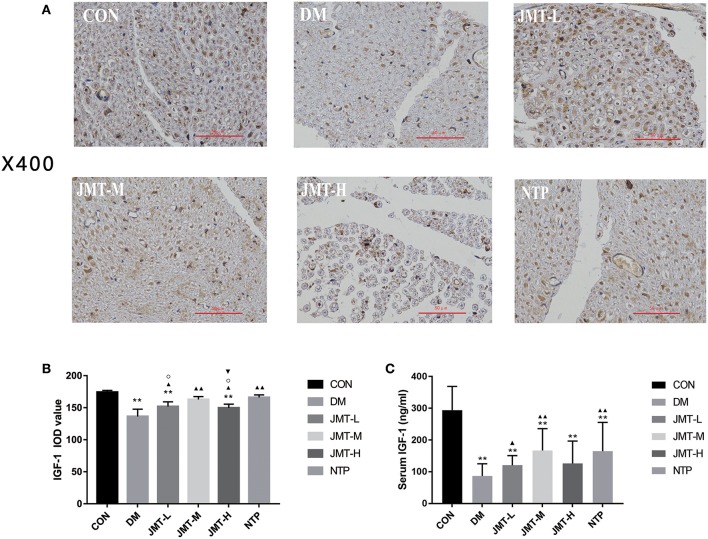
JMT raised the IGF-1 expression in the serum and sciatic nerve of the DPN rats. **(A)** IGF-1 immunohistochemistry of cross section slices of sciatic nerve in Control, DM, JMT-L, JMT-M, JMT-H, and NTP groups, respectively. Immunohistochemical staining images were viewed at a magnification of 400. **(B)** Relative optical density of IGF-1 in different groups detected by immunohistochemistry. **(C)** Serum IGF-1 level in different groups measured by ELISA analysis. **P < 0.01 vs. Con group; ^▴^*P* < 0.05 vs. DM group, ^▴▴^*P* < 0.01 vs. DM group; ^▾^*P* < 0.05 vs. JMT-M group; °*P* < 0.05 vs. NTP group.

The serum IGF-1 expression in DM group and all treatment groups were all decreased compared with the control group (*P* < 0.01). The serum IGF-1 expression in JMT-L, JMT-M, and NTP groups were increased compared with the DM group (*P* < 0.05). There were no statistical significance between JMT-H and DM group (*P* > 0.05). There were also no statistical significance among all treatment groups (*P* > 0.05) (Supplementary Table 6 and Figure 4). Immunohistochemical staining results showed that IGF-1 protein was mainly distributed in cytoplasm and blood vessels in the sciatic nerve. The IGF-1 expression was decreased in DM group compared with the control group (*P* < 0.01). The IGF-1 expression in all treatment groups were increased compared with the DM group (*P* < 0.05). The IGF-1 expression in JMT-H group was decreased compared with JMT-M group. The IGF-1 expression in JMT-L and JMT-H group was decreased compared with NTP group. There was no statistical significance between JMT-M and NTP group (*P* > 0.05) (Figure 4).

### JMT Up-Regulated the IGF-1, IGF-1R, P0, and PMP22 mRNA Expression in the Sciatic Nerve of the DPN Rats

**Figure 5:**
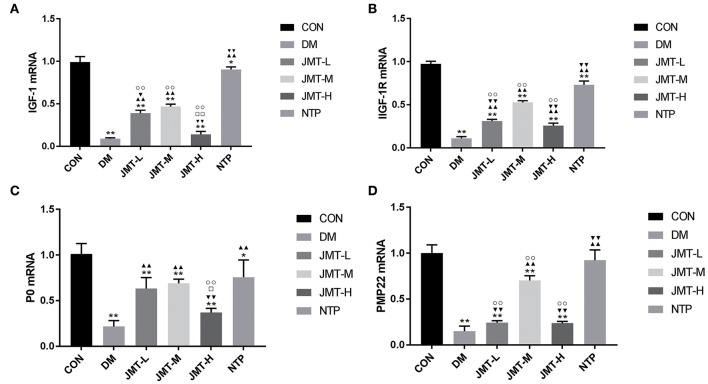
JMT up-regulated the IGF-1, IGF-1R, P0, and PMP22 mRNA expression in the sciatic nerve of the DPN rats. **(A)** IGF-1 mRNA expression in different groups. **(B)** IGF-1R mRNA expression in different groups. **(C)** P0 mRNA expression in different groups. **(D)** PMP22 mRNA expression in different groups. *P < 0.05 vs. Con group, **P < 0.01 vs. Con group; ^▴▴^*P* < 0.01 vs. DM group; ^▾^*P* < 0.05 vs. JMT-M group, ^▾▾^*P* < 0.01 vs. JMT-M group; ^□^*P* < 0.05 vs. JMT-L group, ^□□^*P* < 0.01 vs. JMT-L group; °°*P* < 0.01 vs. NTP group.

The DM group and all treatment groups exhibited obvious decrease in IGF-1, IGF-1R, P0, and PMP22 mRNA expression levels compared with the Con group (*P* < 0.05), except the NTP group showed no difference on the PMP22 mRNA expression with control group (*P* > 0.05). Compared with the DM group, the IGF-1 mRNA expression levels were increased in all treatment group (*P* < 0.01) except JMT-H group (*P* > 0.05), the IGF-1R mRNA expression levels were increased in all treatment group (*P* < 0.01). The IGF-1 mRNA and IGF-1R mRNA expression levels in JMT treatment groups were lower compared with the NTP group. Among the treatment groups, JMT-M group showed the highest IGF-1 mRNA and IGF-1R mRNA expression level. Compared with the DM group, the P0 and PMP22 mRNA expression were increased in JMT-M and NTP group. The P0 and PMP22 mRNA expression level in JMT treatment groups were not higher than NTP groups. Among the JMT treatment groups, the P0 and PMP22 mRNA expression level in JMT-L and JMT-H groups were not higher than JMT-M group (Supplementary Table 7 and Figure 5).

### JMT Up-Regulated the IGF-1, p-IGF-1R, P0, and PMP22 Protein Expression in the Sciatic Nerve of the DPN Rats

**Figure 6:**
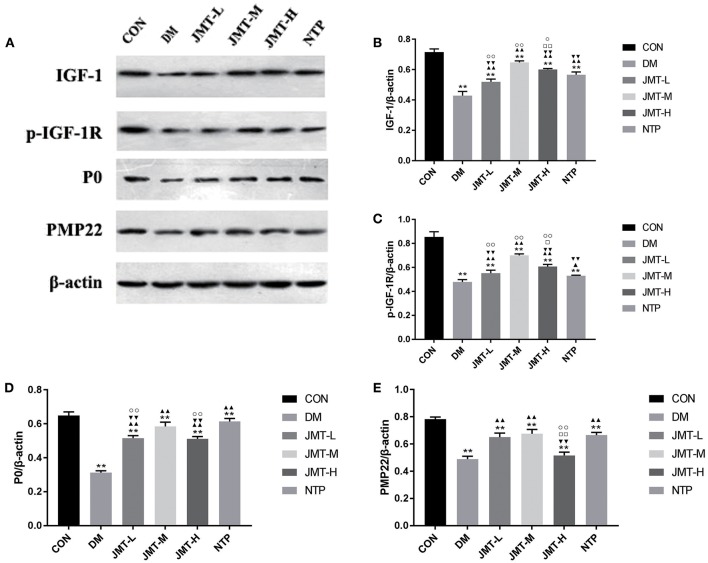
JMT up-regulated the IGF-1, p-IGF-1R, P0, and PMP22 protein expression in the sciatic nerve of the DPN rats. **(A)** The IGF-1, p-IGF-1R, P0, and PMP22 protein expression level measured by Western blot analysis, normalized to β-actin in Con, DM, JMT-L, JMT-M, JMT-H, NTP groups, respectively. **(B)** Relative optical density of IGF-1 expression in different groups. **(C)** Relative optical density of p-IGF-1R expression in different groups. **(D)** Relative optical density of P0 expression in different groups. **(E)** Relative optical density of PMP22 expression in different groups. **P < 0.01 vs. Con group; ^▴^*P* < 0.05 vs. DM group, ^▴▴^*P* < 0.01 vs. DM group; ^▾^*P* < 0.05 vs. JMT-M group, ^▾▾^*P* < 0.01 vs. JMT-M group; ^□^*P* < 0.05 vs. JMT-L group, ^□□^*P* < 0.01 vs. JMT-L group; °*P* < 0.05 vs. NTP group, °°*P* < 0.01 vs. NTP group.

The DM group exhibited a significant decrease in IGF-1, p-IGF-1R, P0, and PMP22 protein expression levels compared with the Con group (*P* < 0.05). Compared with the DM group, the IGF-1, p-IGF-1R, and P0 protein expression levels were increased in each treatment group (*P* < 0.05). Compared with DM group, the PMP22 protein expression levels were increased in each treatment group (*P* < 0.01) except the JMT-H group (*P* > 0.05). The IGF-1 and IGF-1R expression level in JMT-M and JMT-H groups were increased compared with the NTP group (*P* < 0.05). The P0 and PMP22 expression level in JMT treatment groups were not higher than NTP group. Among the treatment groups, JMT-M showed a highest IGF-1 and IGF-1R expression compared with JMT-L and JMT-H groups (*P* < 0.01). The P0 and PMP22 expression level in JMT-L and JMT-H group were not higher than JMT-M group (Figure 6).

## Discussion

In this study, the chemical profile of JMT was firstly characterized and a total of 72 compounds were identified by UPLC/Q-TOF-MS analysis, which may provide the basis for further screening of bioactive components in JMT. Then the neuroprotective effect of JMT was investigated on STZ-induced DM rats. All of the DM rats developed into hyperglycemia with a declining body weight and mechanical withdrawal threshold. The morphology of the sciatic nerves in DM rats was also severely destroyed. On the molecular biology level, the serum IGF-1 concentration of DM rats was significantly reduced, and the expression of IGF-1, p-IGF-1R, P0, and PMP22 protein and corresponding mRNA in the sciatic nerve of the DM rats was also inhibited, suggesting that the peripheral nerve tissue myelin regeneration disorder in DM rats was associated with the inhibition of IGF-1 and its receptor IGF-1R. Different dosage of JMT and NTP can ameliorate the hyperglycemia in DM rats and reverse the behavior results and morphology of DM rats. Besides, the treatments can increase the expression of IGF-1 in serum and improve the IGF-1, IGF-1R, P0, and PMP22 mRNA and protein expressions. As a positive control, the NTP group showed a most significant effect among all the treatments. JMT-M group showed a prominent effect among the three dosage of JMT group.

JMT is a Traditional Chinese Compound Prescription used in the department of traditional Chinese medicine in PUMCH. It is formulated according to the basic theory of traditional Chinese medicine (Shi et al., 2013). Semen Cuscutae nourish yang of the kidney; Fructus Ligustri Lucidi and Herba Ecliptae nourish yin of the kidney and liver; Hirudo and Scorpion improve blood flow and expel stasis; Rhizoma Corydalis relieves pain; Herba Prunella vulgaris and Semen Litchi and resolve hard masses in the body; Ramulus Cinnamoml and Herba Asarum warm and unblock channels and vessels, and promote qi and blood circulation in the body; Semen Persicae and Semen Cassiae relieve constipation (Yin et al., 2015). Accumulated evidences have shown that these drugs exhibited a number of biological properties including anti-oxidative, anti-inflammation, anti-diabetes, neuroprotective effects and so on, according to the pharmacology of Chinese Materia Medica.

We putatively identified 72 chemical constitutes in JMT by the UPLC/Q-TOF-MS analysis. Among them, luteoloside, ecliptasaponin A, isochlorogenic acid C, and isoquercitrin are identified from Herba Ecliptae. They were reported with anti-microbial, anti-inflammation (Rogerio et al., 2007; Wang et al., 2018), preventing collagen deposition, and pulmonary interstitial fibrosis (You et al., 2015), antioxidant (Jung et al., 2010), anti-cancer (Huang et al., 2014), and antidiabetic (Huang et al., 2017) pharmacological activities. Isochlorogenic acid C has been used as a remedy for the treatment of diabetes and its associated complications (Chang et al., 2015). Asarinin comes from Herba Asarum, which has anti-oxidative function and induces specifically cancer cell apoptotic death (Park et al., 2017; Jeong et al., 2018). Rhein, chrysophanol, and aurantio-obstusin were identified from Semen Cassiae. Studies highlight their effects on improving insulin secretion (Du et al., 2012), enhancing insulin-dependent glucose transport (Lee and Sohn, 2008), anti-oxidative, anti-hypertension, anti-mutagenic, anti-genotoxic, anti-allergic, and neuroprotective effects (Hou et al., 2018). Hyperoside, astragalin, quercetin, isoquercitrin, and kaempferol were all identified from Semen Cuscutae. They were reported with neuroprotective, antioxidative, anti-inflammatory, and antidiabetic effects (Ke et al., 2012; Zhang et al., 2013; Ku et al., 2014; Alkhalidy et al., 2018). Quercetin has been reported as an effective therapeutic for type two diabetes mellitus (T2DM) and exerted excellent properties in islet protection and amelioration in animal experiments (Zhuang et al., 2018). Salidroside and nuezhenoside were identified from Fructus Ligustri Lucidi. They were reported with potent antioxidant (Li et al., 2007), anti-viral, neuroprotective (Qu et al., 2012), hepatoprotective, and antidiabetic effects (Yu et al., 2007; Ju et al., 2017). Oleanolic acid and ursolic acid derived from Fructus Ligustri Lucidi and Herba Prunella vulgaris. In particular, the benefits of their effects has been well-accepted on prevention and treatment of T2DM and its associated complications, such as non-alcoholic fatty liver disease, nephropathy, retinopathy, atherosclerosis, and other metabolic syndrome (Camer et al., 2014; Wang et al., 2015). Based on these literatures and our results, we speculated that JMT might exert the anti-diabetes and neuroprotection effect by absorption and metabolism of these active constituents *in vivo*.

Recent studies show that IGF-1 has a hypoglycemic response similar to insulin and is capable of modulating insulin receptor (IR) activities. Moreover, genetic studies demonstrate that low IGF-1 is associated with both T1DM and T2DM and decreased IGF-1 increases the risk of glucose intolerance and T2DM (Huffman et al., 2017). IGF-1 not only plays an important role in the development of T2DM (Rajpathak et al., 2009), but also is a pluripotent growth factor with multiple functions in the peripheral and central nervous system. It supports neuronal survival and axon growth, and also acts on myelinating Schwann cells and oligodendroglia, which is an important stimulus for myelin membrane formation (Liang et al., 2007; Nageeb et al., 2018). Rauskolb found the expression of IGF-1 and its corresponding receptor IGF-1R are dysregulated in patients with diabetes and neurodegenerative diseases. Motor axon degeneration was also observed in mice in which IGF-1R was conditionally depleted in motoneurons, indicating that IGF-1 on IGF-1R activity is important in motoneurons. Studies imply that sensory complications of diabetic polyneuropathy can be corrected by raising circulating IGF-1 levels to a physiological standard by either a gene or a protein therapeutic, or by agents that can serve to up-regulate IGF-1 without correcting hyperglycemia (Rauskolb et al., 2017). As a traditional Chinese compound prescription, JMT showed promising ability in regulating the gene and protein levels of IGF-1 and IGF-1R in DPN rat models, which is consistent with the current studies on the beneficial effects of IGF-1 on DPN. This study focused on discussing the relationship between IGF-1 and its receptor IGF-1R with myelin related genes P0 and PMP22 in JMT-treated DPN rats, which further illustrated the mechanism of JMT on the regulation of IGF-1 binding to its receptor and further enhance the myelin related gene expression. These results verified a new neuroprotection mechanism of JMT by targeting IGF-1 and IGF-1R, which might provide more scientific evidence for the clinical application of the middle dose of JMT in DPN patients.

The existing problems and future study directions were also summarized as follows; firstly, because of the complicated composition of JMT, only parts of major compounds were identified presently. The key effective constituents remain unknown. Secondly, a group of IGF-1 knock out rats could be added as a negative control. Thirdly, more detection methods could be added in behavior study, such as the cold immersion test and hot immersion test to detect the thermal latency. Fourthly, the nerve conduction velocity should be detected as the golden standard for DPN. In addition, it will be much better if this study could find the IGF-1 and IGF-1R downstream signal pathways and define the target proteins or genes the JMT regulate. We plan to carry out an intensive study on JMT treating the DPN rat model, with a more rigorous research design and more detailed methods to verify our hypothesis. Transgenic mouse, HPLC–MS/MS analysis technique, behavior studies, electrophysiology, genomics, transcriptomics, proteomics, and metabonomics will be our further research goals.

## Conclusions

In conclusion, the present findings clearly indicate that the middle dosage of JMT could obviously improve the functional and structural of sciatic nerve of DPN rats by increasing serum and sciatic nerve IGF-1 expression. The molecular mechanism of JMT regulation IGF-1 expression is involved in promoting IGF-1 binding to IGF-1R, and activating myelin related gene P0 and PMP22 expression to repair the nerve injury and help nerve regeneration.

## Author Contributions

XL contributed to the concept and design of the study and supervised this research. WS contributed to the HPLC/MS analysis and wrote part of the manuscript. WJ, CW, YS, and LG performed the animal experiments. JX interpreted the results, drafted and wrote the manuscript, and finally approved the submission of this research. LQ and WL were involved in the experiments, and provided direction and guidance during the researching process. XL is responsible for the overall contents. All authors have read and approved the final manuscript.

### Conflict of Interest Statement

The authors declare that the research was conducted in the absence of any commercial or financial relationships that could be construed as a potential conflict of interest.
