# Supplementary material for: Jinmaitong, a Traditional Chinese Compound Prescription, Ameliorates the Streptozocin-Induced Diabetic Peripheral Neuropathy Rats by Increasing Sciatic Nerve IGF-1 and IGF-1R Expression
Source: Front Pharmacol. 2019 Mar 29;10:255. doi: 10.3389/fphar.2019.00255 (PMC6450141; doi:10.3389/fphar.2019.00255)
Supplement: Supplementary file 1 [file Table_1.docx]

**Supplementary Table 1|** Detailed information of the crude drugs composed in JMT.

| **No.** | **Drug Name** | **Authentication** | **Voucher specimen** |
| --- | --- | --- | --- |
| jmt15-A | Semen Cuscutae | seeds of *Cuscuta chinensis* Lam. | 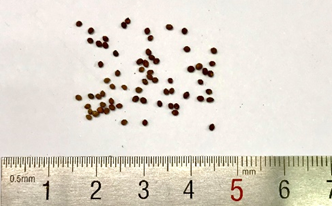 |
| jmt15-B | Fructus Ligustri lucidi | seeds of *Ligustrum lucidum* Ait. | 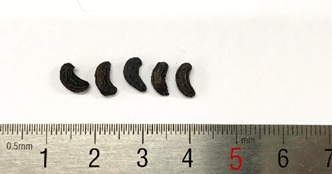 |
| jmt15-C | Herba Ecliptae | whole herb of *Eclipta prostrata* L. | 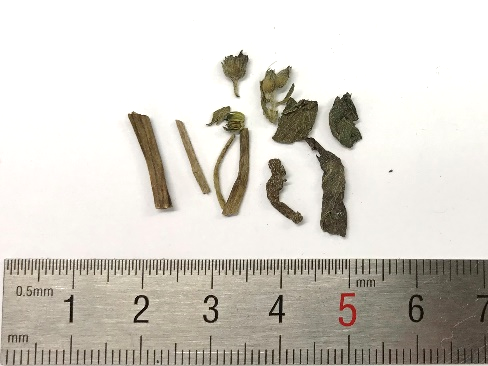 |
| jmt15-D | Herba Prunella vulgaris | whole herb of *Prunella vulgaris* L. | 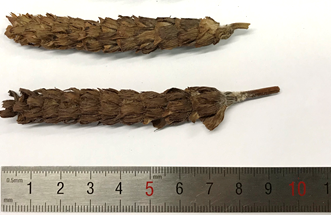 |
| jmt15-E | Semen Litchi | seeds of *Litchi chinensis* Sonn. | 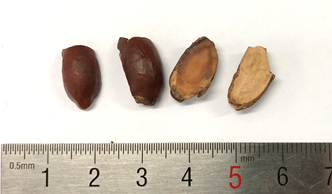 |
| jmt15-F | Scorpio | *Buthus martensii* K. | 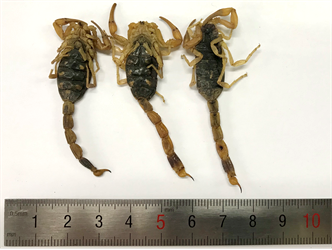 |
| jmt15-G | Ramulus Cinnamoml | tender stem of *Cinnamomum cassia* Presl. | 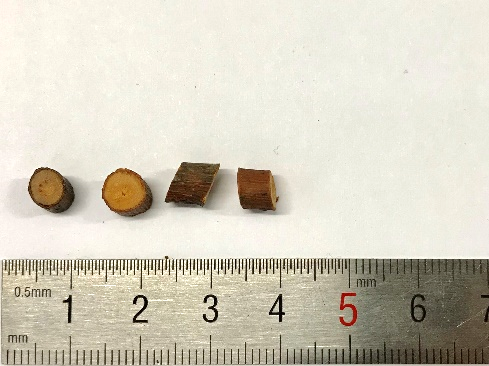 |
| jmt15-H | Rhizoma Corydalis | rhizoma of *Corydalis yanhusuo* W. T. Wang | 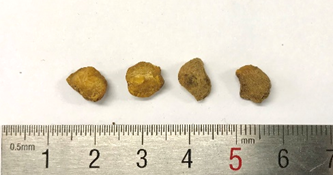 |
| jmt15-I | Semen Persicae | seeds of *Prunus persica* L. | 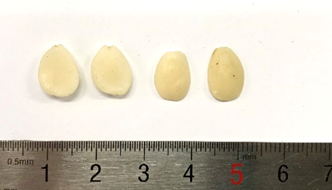 |
| jmt15-J | Semen Cassiae | seeds of *Cassia obtusifolia L.* or *Cassia tora* L. | 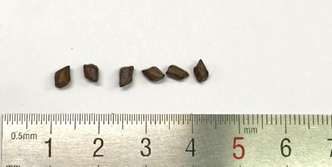 |
| jmt15-K | Radix et Rhizoma Asari | radix and rhizoma of *Asarum heterotropiodes* F. | *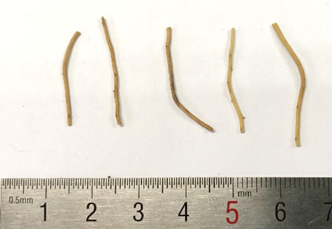* |
| jmt15-L | Hirudo | *Hirudo nipponica*W. | 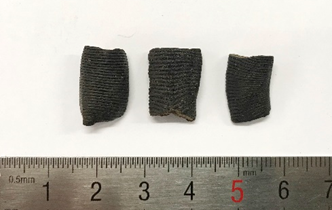 |
