# Supplementary material for: Jinmaitong, a Traditional Chinese Compound Prescription, Ameliorates the Streptozocin-Induced Diabetic Peripheral Neuropathy Rats by Increasing Sciatic Nerve IGF-1 and IGF-1R Expression
Source: Front Pharmacol. 2019 Mar 29;10:255. doi: 10.3389/fphar.2019.00255 (PMC6450141; doi:10.3389/fphar.2019.00255)
Supplement: Supplementary file 2 [file Table_2.docx]

**Supplementary** **Table 2|** Detailed information of the antibodies used in this study.

| Name of Antibody | Manufacture (Host) | Working Dilution | Applications |
| --- | --- | --- | --- |
| IGF-1 (K149) | bioword(Rabbit) | 1:100 | IHC |
| Goat Anti-Rabbit IgG | abcam (Goat) | 1:1000 | IHC |
| IGF1 | abcam (Mouse) | 1:1000 | WB |
| IGF1 Receptor (phospho Y1161) | abcam (Rabbit) | 1:100 | WB |
| Myelin Protein Zero | abcam (Rabbit) | 1:1000 | WB |
| PMP22 | abcam (Rabbit) | 1:1000 | WB |
| β-actin | abcam (Mouse) | 1:10000 | WB |
| Goat Anti-Rabbit IgG | abcam (Goat) | 1:10000 | WB |
| Goat Anti-Mouse IgG | abcam (Goat) | 1:10000 | WB |
