# Supplementary material for: Jinmaitong, a Traditional Chinese Compound Prescription, Ameliorates the Streptozocin-Induced Diabetic Peripheral Neuropathy Rats by Increasing Sciatic Nerve IGF-1 and IGF-1R Expression
Source: Front Pharmacol. 2019 Mar 29;10:255. doi: 10.3389/fphar.2019.00255 (PMC6450141; doi:10.3389/fphar.2019.00255)
Supplement: Supplementary file 3 [file Table_3.docx]

**Supplementary** **Table 3|** Effect of JMT on fasting blood glucose levels in DPN rats.

| Group | *n* | Pre-treatment(mmol/L) | *P*ost-treatment(mmol/L) | | | |
| --- | --- | --- | --- | --- | --- | --- |
|  |  |  | 4w | 8w | 12w | 16w |
| CON | 10 | 5.2±0.5 | 5.2±0.5 | 5.3±0.2 | 5.6±0.4 | 5.2±1.0 |
| DM | 8 | 23.6±2.7^**^ | 24.9±2.4^**^ | 25.5±2.0^**^ | 26.3±1.5^**^ | 27.3±1.2^**^ |
| JMT-L | 10 | 23.3±2.9^**^ | 24.2±2.8^**^ | 25.2±2.3^**^ | 25.3±1.1^**^ | 24.6±1.9^**▲▲^ |
| JMT-M | 9 | 23.7±3.2^**^ | 24.5±3.0^**^ | 24.8±2.8^**^ | 24.7±2.6^**^ | 24.0±1.8^**▲▲^ |
| JMT-H | 9 | 23.5±2.7^**^ | 24.6±1.8^**^ | 25.0±1.8^**^ | 25.4±1.9^**^ | 24.9±1.5^**▲▲^ |
| NTP | 9 | 23.8±2.3^**^ | 24.3±1.5^**^ | 24.5±2.1^**^ | 24.9±3.0^**^ | 24.1±2.6^**▲▲^ |
| Data are shown as the mean standard deviation. ^**^*P* <0.01 vs. Con group.^▲▲^*P* <0.01 vs. DM group. Con, normal control; DM, diabetic model control; JMT, Jinmaitong; -L, -low-dosage; -M, medium-dosage; -H, -high-dosage; NTP, Neurotropin. | | | | | | |
