# Supplementary material for: Jinmaitong, a Traditional Chinese Compound Prescription, Ameliorates the Streptozocin-Induced Diabetic Peripheral Neuropathy Rats by Increasing Sciatic Nerve IGF-1 and IGF-1R Expression
Source: Front Pharmacol. 2019 Mar 29;10:255. doi: 10.3389/fphar.2019.00255 (PMC6450141; doi:10.3389/fphar.2019.00255)
Supplement: Supplementary file 4 [file Table_4.docx]

**Supplementary** **Table 4|** Effect of JMT on body weight in DPN rats.

| Group | *n* | Pre-treatment (g) | Post-treatment (g) | | | |
| --- | --- | --- | --- | --- | --- | --- |
|  |  |  | 4w | 8w | 12w | 16w |
| CON | 10 | 265±10 | 434±28 | 482±33 | 523±33 | 552±34 |
| DM | 8 | 239±20^**^ | 301±28^**^ | 310±29^**^ | 315±32^**^ | 317±32^**^ |
| JMT-L | 10 | 238±17^**^ | 315±37^**^ | 323±35^**^ | 328±32^**^ | 329±34^**^ |
| JMT-M | 9 | 241±13^**^ | 319±41^**^ | 325±38^**^ | 330±35^**^ | 335±40^**^ |
| JMT-H | 9 | 240±21^**^ | 303±22^**^ | 316±26^**^ | 321±22^**^ | 323±22^**^ |
| NTP | 9 | 239±18^**^ | 309±23^**^ | 326±22^**^ | 332±20^**^ | 340±22^**^ |
| Data are shown as the mean standard deviation. ^**^*P* <0.01 vs. Con group. Con, normal control; DM, diabetic model control; JMT, Jinmaitong; -L, -low-dosage; -M, medium-dosage; -H, -high-dosage; NTP, Neurotropin. | | | | | | |
