# Supplementary material for: Jinmaitong, a Traditional Chinese Compound Prescription, Ameliorates the Streptozocin-Induced Diabetic Peripheral Neuropathy Rats by Increasing Sciatic Nerve IGF-1 and IGF-1R Expression
Source: Front Pharmacol. 2019 Mar 29;10:255. doi: 10.3389/fphar.2019.00255 (PMC6450141; doi:10.3389/fphar.2019.00255)
Supplement: Supplementary file 5 [file Table_5.docx]

**Supplementary** **Table 5|** Effect of JMT on mechanical pain threshold in DPN rats.

| Group | *n* | Left Value (g) | Right Value (g) |
| --- | --- | --- | --- |
| CON | 10 | 82.8±15.5 | 78.6±16.7 |
| DM | 8 | 34.3±10.7^**^ | 33.0±10.9^**^ |
| JMT-L | 10 | 60.5±11.0^**^^▲▲^^○^ | 56.7±12.8^**▲▲^ |
| JMT-M | 9 | 65.6±11.7^**▲▲^^○○^ | 63.3±13.0^**▲▲○○^ |
| JMT-H | 9 | 43.4±12.2^**▲^^▼▼□□○^ | 46.7±10.8^**▲▲▼▼□□○^ |
| NTP | 9 | 52.6±18.2^**▲▲▼▼^ | 53.7±13.1^**▲▲▼▼^ |
| Data are shown as the mean ± standard deviation. ^**^*P* <0.01 vs. Con group; ^▲^*P* <0.05 vs. DM group, ^▲▲^*P* <0.01 vs. DM group; ^▼▼^*P* <0.01 vs. JMT-M group; ^□□^*P* <0.01 vs. JMT-L group; ^○^*P* <0.05 vs. NTP group, ^○○^*P* <0.01 vs. NTP group. Con, normal control; DM, diabetic model control; JMT, Jinmaitong; -low-dosage; -M, medium-dosage; -H, -high-dosage; NTP, Neurotropin. | | | |
