# Supplementary material for: Jinmaitong, a Traditional Chinese Compound Prescription, Ameliorates the Streptozocin-Induced Diabetic Peripheral Neuropathy Rats by Increasing Sciatic Nerve IGF-1 and IGF-1R Expression
Source: Front Pharmacol. 2019 Mar 29;10:255. doi: 10.3389/fphar.2019.00255 (PMC6450141; doi:10.3389/fphar.2019.00255)
Supplement: Supplementary file 6 [file Table_6.docx]

**Supplementary** **Table 6|** The serum IGF-1 expression in different groups.

| Groups | n | IGF-1 |
| --- | --- | --- |
| CON | 6 | 293.99±74.91 |
| DM | 6 | 86.74±38.30^**^ |
| JMT-L | 6 | 120.38±30.60^**▲^ |
| JMT-M | 6 | 167.01±68.76^**^^▲▲^ |
| JMT-H | 6 | 126.11±70.59^**^ |
| NTP | 6 | 164.68±90.66^**▲▲^ |
| Data are shown as the mean ± standard deviation. ^**^*P* <0.01 vs. Con group; ^▲^*P* <0.05 vs. DM group, ^▲▲^*P* <0.01 vs. DM group. | | |
