# Supplementary material for: Jinmaitong, a Traditional Chinese Compound Prescription, Ameliorates the Streptozocin-Induced Diabetic Peripheral Neuropathy Rats by Increasing Sciatic Nerve IGF-1 and IGF-1R Expression
Source: Front Pharmacol. 2019 Mar 29;10:255. doi: 10.3389/fphar.2019.00255 (PMC6450141; doi:10.3389/fphar.2019.00255)
Supplement: Supplementary file 7 [file Table_7.docx]

**Supplementary** **Table 7|** IGF-1, IGF-1R, P0 and PMP22 mRNA expression in different groups.

| Groups | IGF-1 | IGF-1R | P0 | PMP22 |
| --- | --- | --- | --- | --- |
| CON | 0.991±0.065 | 0.970±0.031 | 1.011±0.111 | 0.997±0.090 |
| DM | 0.090±0.011^**^ | 0.111±0.019^**^ | 0.215±0.069^**^ | 0.150±0.053^**^ |
| JMT-L | 0.390±0.034^**^^▲▲▼○○^ | 0.310±0.022^**▲▲▼▼○○^ | 0.632±0.122^**^^▲▲^ | 0.246±0.021^**^^▼▼^^○○^ |
| JMT-M | 0.466±0.032^**▲▲○○^ | 0.527±0.018^**▲▲○○^ | 0.688±0.046^**▲▲^ | 0.704±0.052^**^^▲▲○○^ |
| JMT-H | 0.142±0.038^**^^▼▼□□^^○○^ | 0.257±0.032^**▲▲▼▼○○^ | 0.371±0.045^**▼▼□^^○○^ | 0.236±0.021^**▼▼○○^ |
| NTP | 0.903±0.029^*▲▲▼▼^ | 0.730±0.047^**▲▲▼▼^ | 0.759±0.190^*▲▲^ | 0.924±0.111^▲▲▼▼^ |
| Data are shown as the mean ± standard deviation. ^*^*P* <0.05 vs. Con group, ^**^*P* <0.01 vs. Con group; ^▲▲^*P* <0.01 vs. DM group; ^▼^*P* <0.05 vs. JMT-M group, ^▼▼^*P* <0.01 vs. JMT-M group; ^□^*P* <0.05 vs. JMT-L group; ^○○^*P* <0.01 vs. NTP group. | | | | |
